# Supplementary material for: Technical development and In Silico implementation of SyntheticMR in head and neck adaptive radiation therapy: A prospective R‐IDEAL stage 0/1 technology development report
Source: J Appl Clin Med Phys. 2025 Jul 11;26(7):e70134. doi: 10.1002/acm2.70134 (PMC12257339; doi:10.1002/acm2.70134)
Supplement: Supplementary file 1 — Supporting Information [file ACM2-26-e70134-s001.docx]

**Supporting Material**


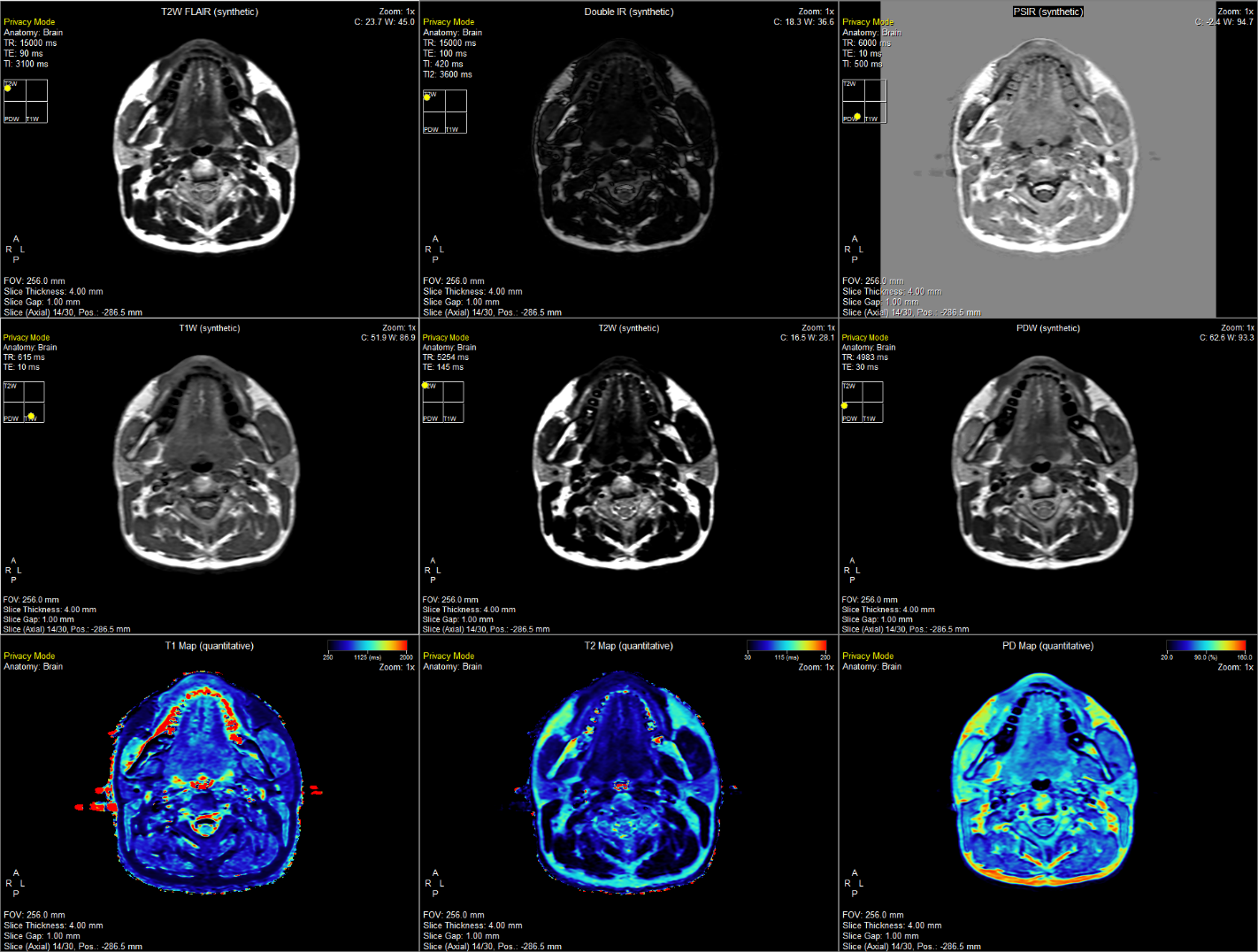


Figure 3-S1. Demonstration of the SyMRI post-processing package offered by SyntheticMR in Volunteer 1 on the MR-Sim scanner.


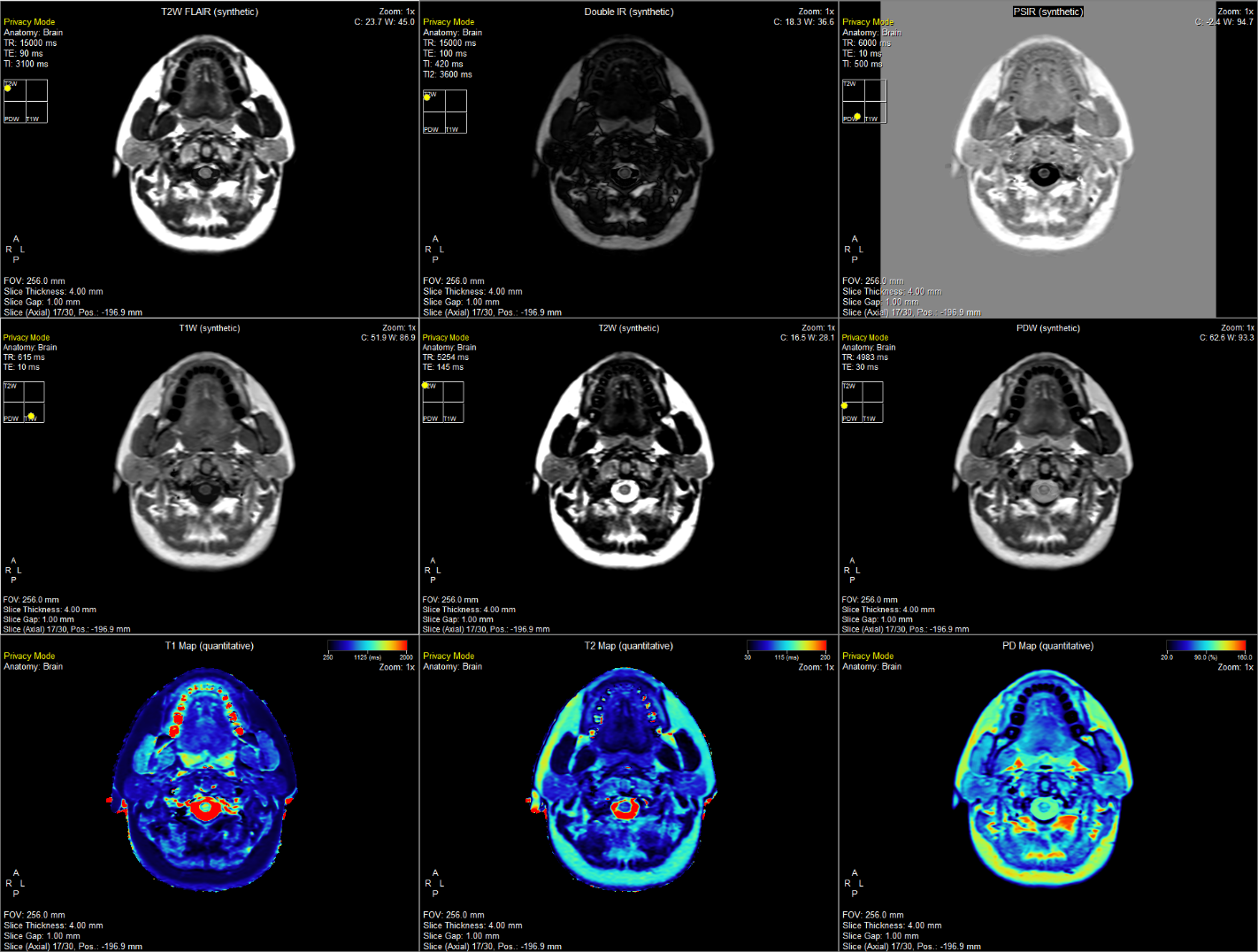


Figure 3-S2. Demonstration of the SyMRI post-processing package offered by SyntheticMR in Volunteer 2 on the MR-Sim scanner.


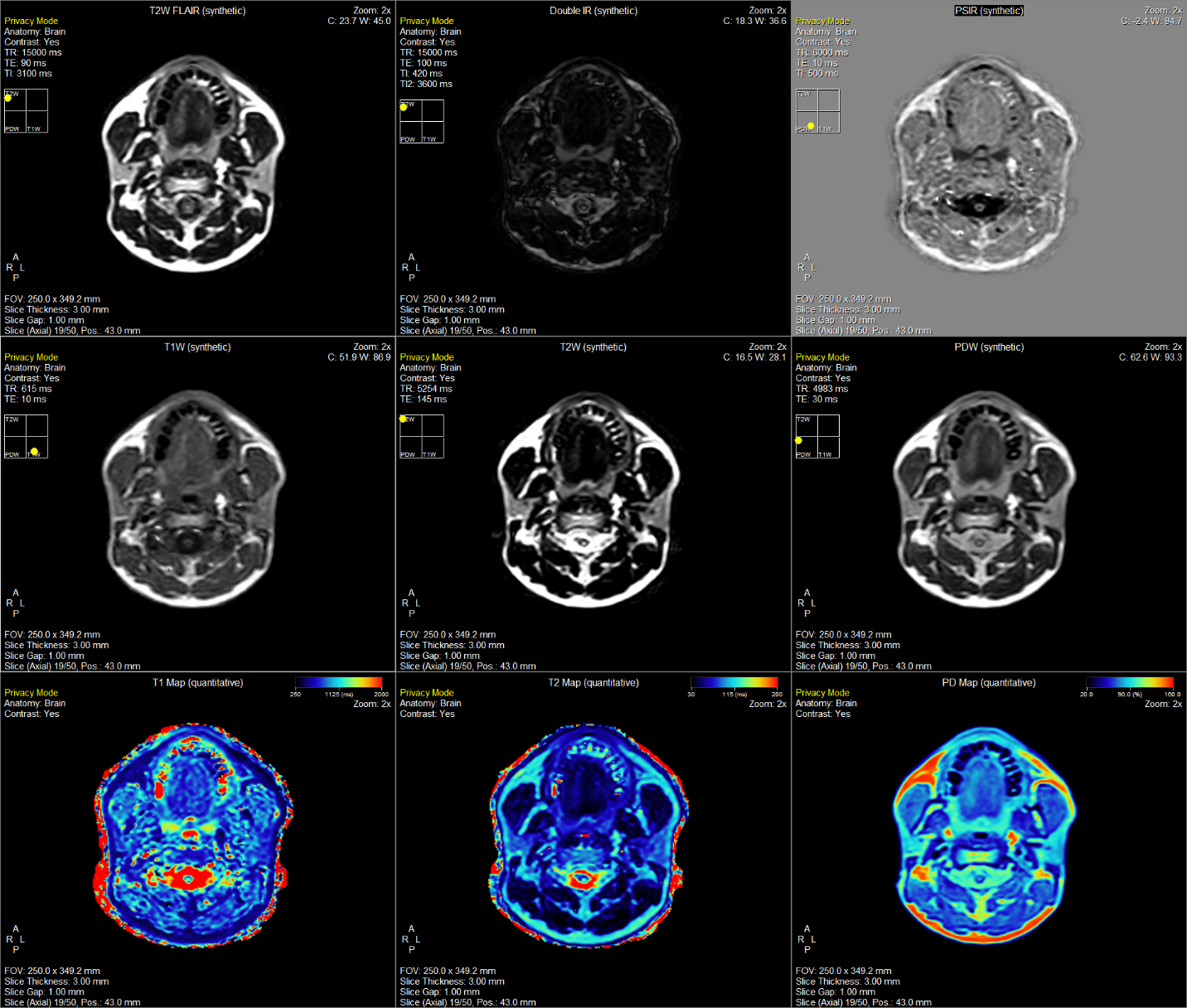


Figure 3-S3. Demonstration of the SyMRI post-processing package offered by SyntheticMR in Volunteer 1 using the coarse sequence on the MR-Linac scanner.


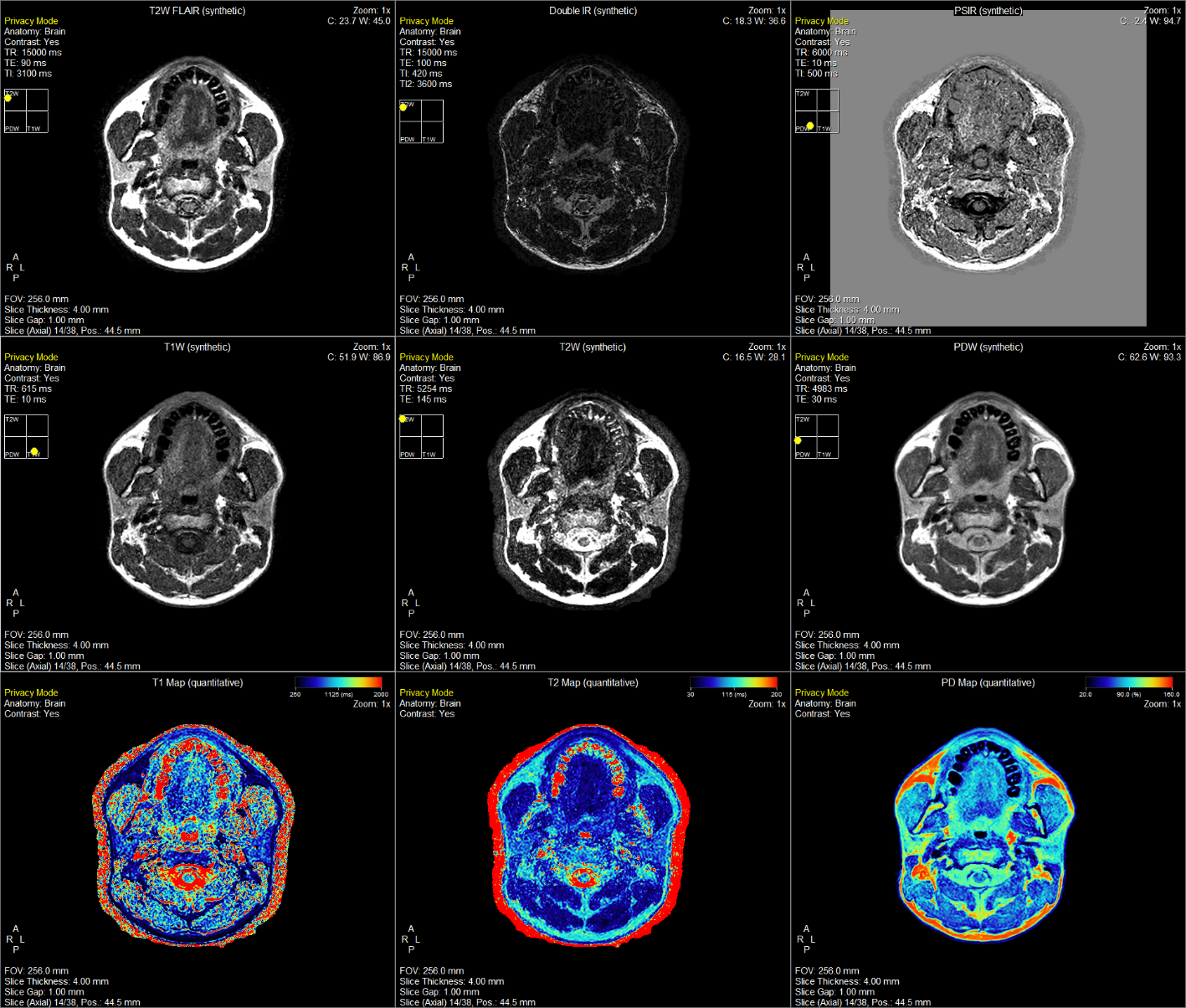


Figure 3-S4. Demonstration of the SyMRI post-processing package offered by SyntheticMR in Volunteer 1 using the fine sequence on the MR-Linac scanner.

| Volunteer 1  MR-Sim | 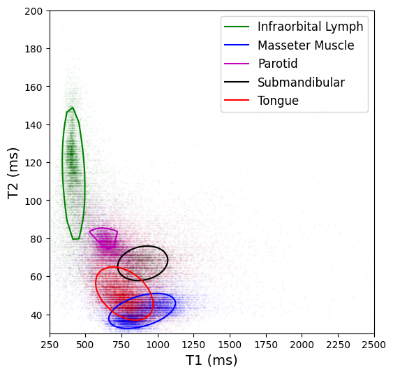 | 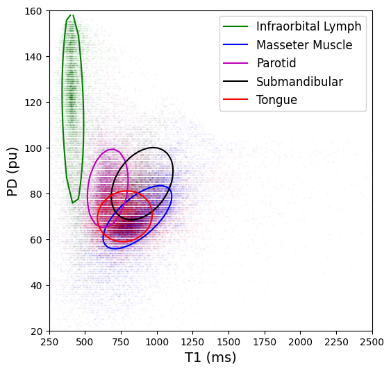 | 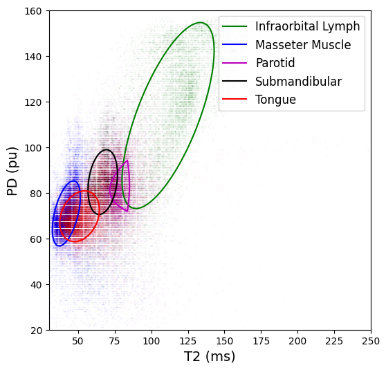 |
| --- | --- | --- | --- |
| Volunteer 2  MR-Sim | 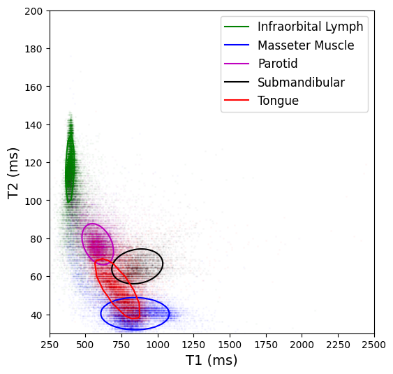 | 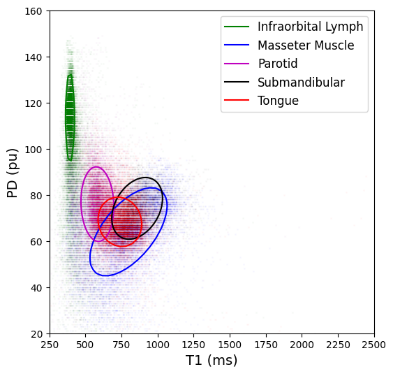 | 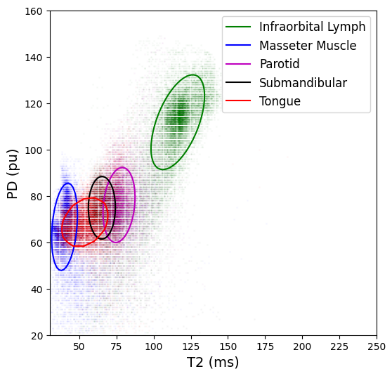 |
| Volunteer 1  MR-Linac (coarse) | 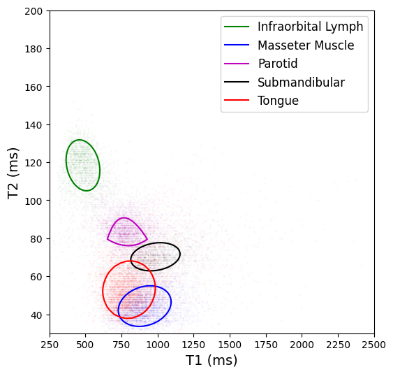 | 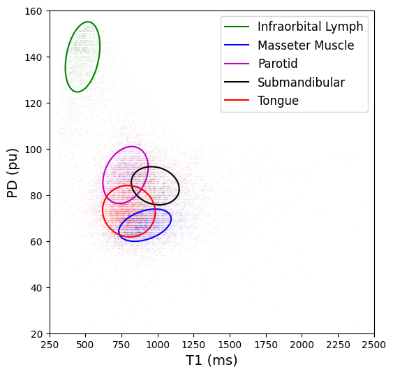 | 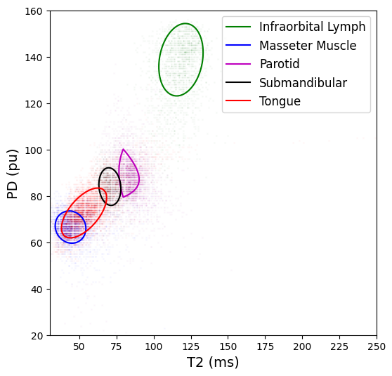 |
| Volunteer 1  MR-Linac (fine) | 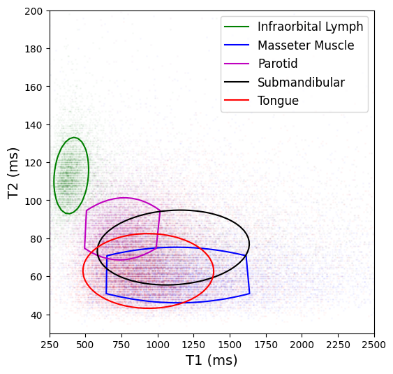 | 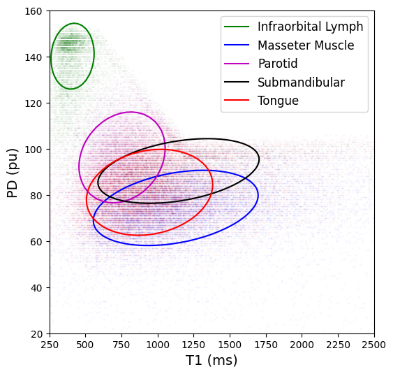 | 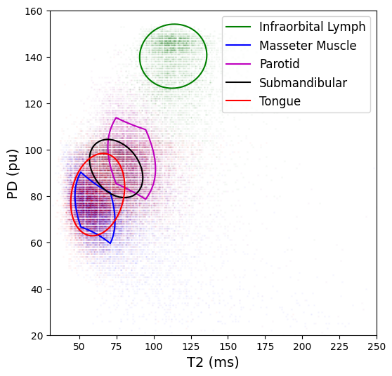 |

Figure 4-S1. Cluster analysis of normal tissue values for each combination of quantitative parameters (i.e., T1, T2, and PD) on both the MR-Sim and MR-Linac.
